# Isotopic effects on in-plane hyperbolic phonon polaritons in MoO_3_

**DOI:** 10.1515/nanoph-2023-0717

**Published:** 2024-03-04

**Authors:** Jeremy F. Schultz, Sergiy Krylyuk, Jeffrey J. Schwartz, Albert V. Davydov, Andrea Centrone

**Affiliations:** Physical Measurement Laboratory, National Institute of Standards and Technology, Gaithersburg, MD 20899, USA; Material Measurement Laboratory, National Institute of Standards and Technology, Gaithersburg, MD 20899, USA; Department of Electrical and Computer Engineering, University of Maryland, College Park, MD 20742, USA

**Keywords:** 2D materials, hyperbolic materials, isotopic engineering, phonon polaritons, photothermal induced resonance

## Abstract

Hyperbolic phonon polaritons (HPhPs), hybrids of light and lattice vibrations in polar dielectric crystals, empower nanophotonic applications by enabling the confinement and manipulation of light at the nanoscale. Molybdenum trioxide (α-MoO_3_) is a naturally hyperbolic material, meaning that its dielectric function deterministically controls the directional propagation of in-plane HPhPs within its reststrahlen bands. Strategies such as substrate engineering, nano- and hetero-structuring, and isotopic enrichment are being developed to alter the intrinsic dielectric functions of natural hyperbolic materials and to control the confinement and propagation of HPhPs. Since isotopic disorder can limit phonon-based processes such as HPhPs, here we synthesize isotopically enriched ^92^MoO_3_ (^92^Mo: 99.93 %) and ^100^MoO_3_ (^100^Mo: 99.01 %) crystals to tune the properties and dispersion of HPhPs with respect to natural α-MoO_3_, which is composed of seven stable Mo isotopes. Real-space, near-field maps measured with the photothermal induced resonance (PTIR) technique enable comparisons of in-plane HPhPs in α-MoO_3_ and isotopically enriched analogs within a reststrahlen band (≈820 cm^−1^ to ≈972 cm^−1^). Results show that isotopic enrichment (e.g., ^92^MoO_3_ and ^100^MoO_3_) alters the dielectric function, shifting the HPhP dispersion (HPhP angular wavenumber × thickness vs. IR frequency) by ≈−7 % and ≈+9 %, respectively, and changes the HPhP group velocities by ≈±12 %, while the lifetimes (≈3 ps) in ^92^MoO_3_ were found to be slightly improved (≈20 %). The latter improvement is attributed to a decrease in isotopic disorder. Altogether, isotopic enrichment was found to offer fine control over the properties that determine the anisotropic in-plane propagation of HPhPs in α-MoO_3_, which is essential to its implementation in nanophotonic applications.

## 1 Introduction

Polaritons are quasiparticles resulting from the hybridization of light with coherent charge oscillations in a material [1], [2]. These hybrid excitations guide and confine light to dimensions smaller than the optical diffraction limit, leading to enhanced near-fields [3], [4], [5], which enable nonlinear spectroscopies [6] as well as nanophotonic and optoelectronic applications [7], [8], [9], [10]. While surface plasmon polaritons can be engineered across a broad spectral range, they are typically hindered by rapid scattering rates and high losses, resulting in modes with short lifetimes and broad linewidths [11]. By contrast, hyperbolic phonon polaritons (HPhPs) intrinsically exhibit substantially lower optical losses and increased lifetimes but exist only within mid-IR spectral regions of high reflectivity. These spectral regions, known as reststrahlen bands, where at least one component of the electric permittivity tensor is negative and at least one component is positive, are delimited by pairs of transverse optical (TO) and longitudinal optical (LO) phonon modes [12]. Within reststrahlen bands, HPhPs propagate through a material’s volume with angles determined by its frequency-dependent permittivity [13]. Since HPhPs are highly compressed with respect to the free-space wavelength, they are typically characterized with near-field imaging, performed either with the photothermal induced resonance (PTIR) technique [14], [15], or more commonly with scattering-type scanning near-field optical microscopy (s-SNOM) [1], [16]. With both methods they appear as periodic fringes around defects or material discontinuities [1]. The analysis of real-space images of HPhPs is often used to derive the properties and characteristics of HPhPs as a function of the host material’s composition and environment [1], [14], [15], [16], [17].

While certain materials naturally support HPhPs, therefore not requiring the complex fabrication processes necessary to realize hyperbolic metamaterials [18], tailoring their properties is not straightforward. Strategies to modify their optical properties include the fabrication of atomic-scale heterostructures [19], the photoinjection of free carriers into nanoresonators [20], the intercalation of atoms [21], and the introduction of lattice strain [22], among others [23]. Importantly, some methods that rely on post-growth fabrication strategies, such as ion intercalation [21], have so far been found to introduce additional optical losses [24].

Isotopic enrichment can tune and optimize the properties of 2D materials that are governed by phonon-based processes [25], such as thermal conductivity [26] and electron–phonon interactions [27], [28], while preserving or reducing the optical losses of supported HPhPs [25]. Recently, isotopic enrichment of boron was used to tune the dielectric function of hexagonal boron nitride (hBN), significantly improving the propagation lengths and lifetimes of HPhPs [15], [29], [30], [31] with respect to the isotopic-disorder-limited characteristics of naturally abundant hBN [27], [32]. As a polar van der Waals (vdW) crystal, orthorhombic molybdenum trioxide (α-MoO_3_) also supports HPhPs [33]. Furthermore, naturally abundant Mo exists as a mixture of seven stable isotopes: ^92^Mo (15.86 % abundant), ^94^Mo (9.12 %), ^95^Mo (15.70 %), ^96^Mo (16.50 %), ^97^Mo (9.45 %), ^98^Mo (23.75 %), and ^100^Mo (9.62 %) [34]. As a result, Mo isotopic enrichment has the effect of modestly shifting the optical phonon frequencies delimiting its reststrahlen bands [35] and is expected to improve properties limited by isotopic disorder [34], such as thermal conductivity and polariton lifetimes. For example, Mo isotopic enrichment in MoS_2_ yielded ≈50 % higher in-plane thermal conductivity in isotopically pure ^100^MoS_2_ and longer exciton lifetimes (82 ± 14 ps) with respect to its naturally abundant counterpart (30 ± 14 ps) [36]. Additionally, Mo isotopic enrichment in α-MoO_3_ was recently shown to increase the propagation length (≈6 µm) and lifetimes (≈10 ps, up to a ≈1.7× improvement) of in-plane elliptical phonon polaritons (PhPs), which was attributed to increased optical coherence due to reduced random phonon scattering [35].

Here we use PTIR, a scanning probe-based photothermal nanospectroscopic technique [37], [38], [39], [40], to measure and compare real-space absorption maps of in-plane HPhPs in naturally abundant and isotope-enriched α-MoO_3_ (specifically ^92^MoO_3_ and ^100^MoO_3_) exfoliated onto gold-coated glass. Through the analysis of PTIR absorption maps, we identify the effects of isotopic enrichment on the dispersions, group velocities, propagation lengths, and lifetimes of HPhPs, as well as the dielectric function of α-MoO_3_.

## 2 Results and discussion

PTIR measurements combine the capabilities of atomic force microscopy (AFM) with absorption spectroscopy, yielding absorption spectra and maps with ≈10 nm spatial resolution from the infrared (IR) to the visible spectral ranges [38], [41], [42]. In PTIR, the light emitted from a pulsed, wavelength-tunable laser is focused (≈50 µm diameter) on a portion of the sample, centered on the AFM tip. Upon light absorption, the sample is photothermally heated, expanding rapidly and subsequently contracting (typically < 1 µs) as a function of the sample thermal properties [43]. Measurement of the time-domain photothermal expansion and decay of the sample can determine the thermal conductivity of the sample at the nanoscale but requires ultrasensitive, wide-bandwidth optomechanical cavity AFM probes [43], [44]. By contrast, the rapid sample photothermal expansion kicks conventional AFM cantilevers into oscillation. Importantly, the initial AFM cantilever oscillation amplitude is proportional to the initial sample expansion [40], [44], [45] and to the sample’s local absorption coefficient [46], [47]. This proportionality enables the easy comparison of PTIR spectra with far-field spectral databases, aiding material identification at the nanoscale (down to ≈ 10 nm) [48], [49]. With this tip-based detection scheme, PTIR achieves much higher spatial resolution (≈10 nm) [42] than both the optical diffraction limit (≈*λ*/2, where *λ* is the free-space wavelength of light) and the thermal diffusion limit (typically > 1 µm) [50]. In this work, the mid-IR laser pulse frequency is matched to the frequency of one of the AFM cantilever resonance modes, enhancing the PTIR signal proportionally to the cantilever mode’s mechanical quality factor [40], [51]. Throughout the experiments the matching condition is maintained by a phase-locked loop [52]. The PTIR technique has found broad applications in materials science [49], [53], [54], [55], [56], [57], biology [58], [59], and other fields, as discussed in recent reviews [38], [39], [40]. Recently, PTIR was used to characterize HPhPs in naturally abundant hBN resonators [60] and α-MoO_3_ flakes [14], as well as in isotopically enriched hBN flakes [15]. Other applications pertaining to the characterization of 2D materials include the identification of interlayer contaminants in 2D heterostructures [61], the identification of functional groups in graphene [62], and the study of H_2_ intercalation in α-MoO_3_ crystals [57], among others.

**Figure 1:**
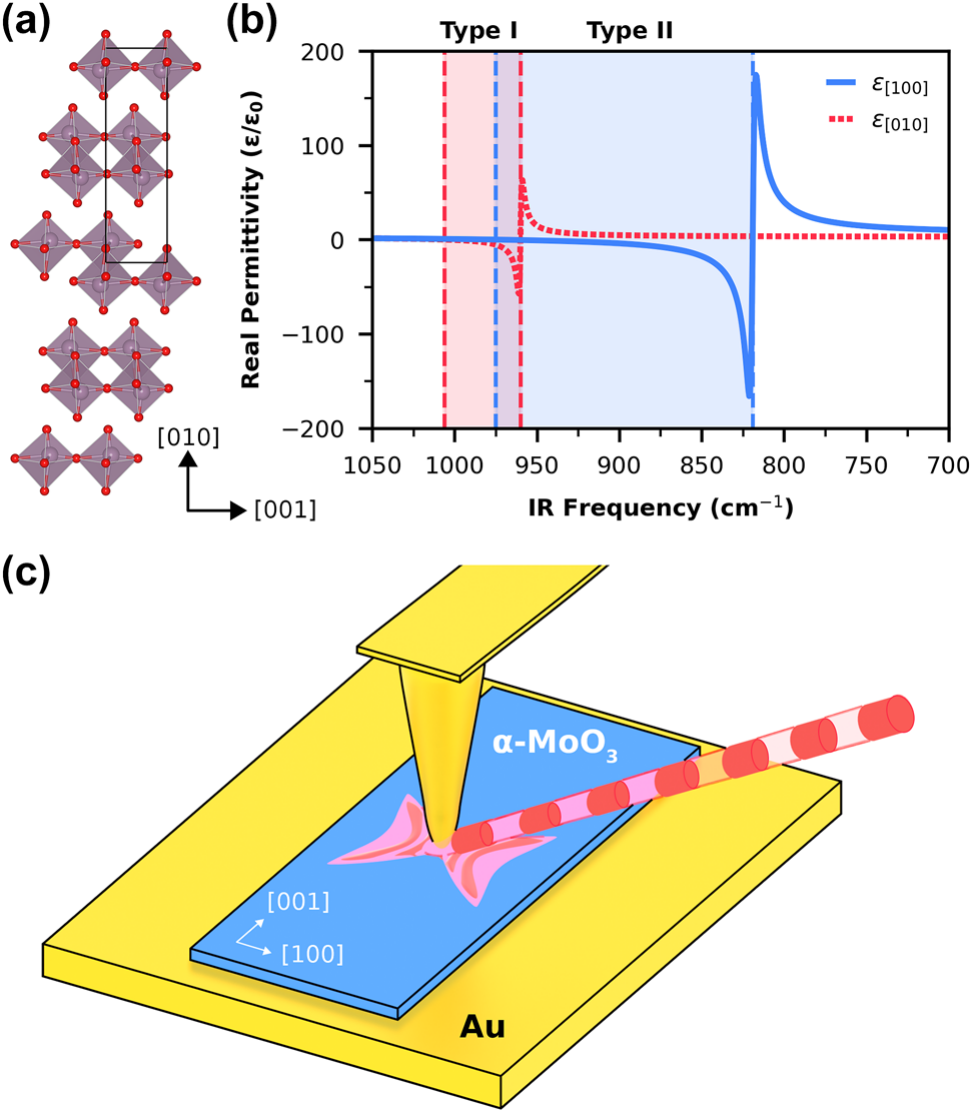
The structure and dielectric properties of α-MoO_3_ along with a schematic of the photothermal induced resonance (PTIR) technique for the detection of hyperbolic phonon polaritons (HPhPs). (a) Crystal structure of α-MoO_3_ viewed along the [100] direction highlighting the unit cell of the van der Waals layered structure. Oxygen atoms are red while Mo atoms are at the center of the gray octahedra. (b) Calculated real components of the complex dielectric function (*ε*) of natural α-MoO_3_ relative to that of free space (*ε*_0_) along the [100] (blue) and [010] (red dashed) crystallographic directions. The highlighted regions indicate two of the reststrahlen bands of α-MoO_3_. The band highlighted in blue where in-plane HPhPs (Type II) occur is the focus of this work. (c) Schematic of the measurement where a metal-coated atomic force microscopy (AFM) tip launches in-plane HPhPs in an α-MoO_3_ crystal deposited on a gold substrate, which are detected via the PTIR technique.

α-MoO_3_ is composed of stacked bilayers consisting of distorted MoO_6_ octahedra, where adjacent layers are held together by vdW interactions [63], resulting in an orthorhombic crystal structure (Figure 1a) with strong optical anisotropy [64], [65]. Due to its layered vdW-type structure, single crystals of α-MoO_3_ can be mechanically exfoliated into thin flakes with well-defined [100] and [001] directions that can be readily identified based on the long and short axes of the flakes [66]. Within its reststrahlen bands, α-MoO_3_ exhibits strong reflectivity with large in-plane anisotropy and supports the propagation of phonon polaritons [33]. The uppermost band (from ≈958 cm^−1^ to ≈1004 cm^−1^, *ε*_[010]_ < 0, *ε*_[100]_ ≠ *ε*_[001]_ > 0) corresponds to a Type I hyperbolic response leading to an elliptical in-plane polariton character. Another reststrahlen band (from ≈820 cm^−1^ to ≈972 cm^−1^; *ε*_[100]_ < 0, *ε*_[010]_ ≠ *ε*_[001]_ > 0) corresponds to a Type II hyperbolic response leading to an in-plane hyperbolic polariton character (Figure 1b).

Due to the momentum mismatch between the incident photons and the HPhPs, excitation of the latter typically requires either evanescent illumination geometries or light-scattering sites [13]. Consequently, HPhPs can be launched from intrinsic features of a material, such as natural or milled crystal edges [67], [68], [69], or from extrinsic features such as a plasmonic launcher [70], [71] or the sharp metal tip of an AFM [72]. Once launched, the highly compressed HPhPs can be visualized with nanoscale resolution near-field techniques such as s-SNOM [16], [73], [74] and PTIR [14], [15]. Since Type II HPhPs possess in-plane hyperbolic dispersions [75], they propagate solely along the [100] direction of α-MoO_3_ flakes (Figure 1c and Figure S1). However, it bears mentioning that recent work has found a high degree of geometrical confinement [76] or an induced optical topological transition [77] can guide HPhPs along otherwise forbidden directions.

**Figure 2:**
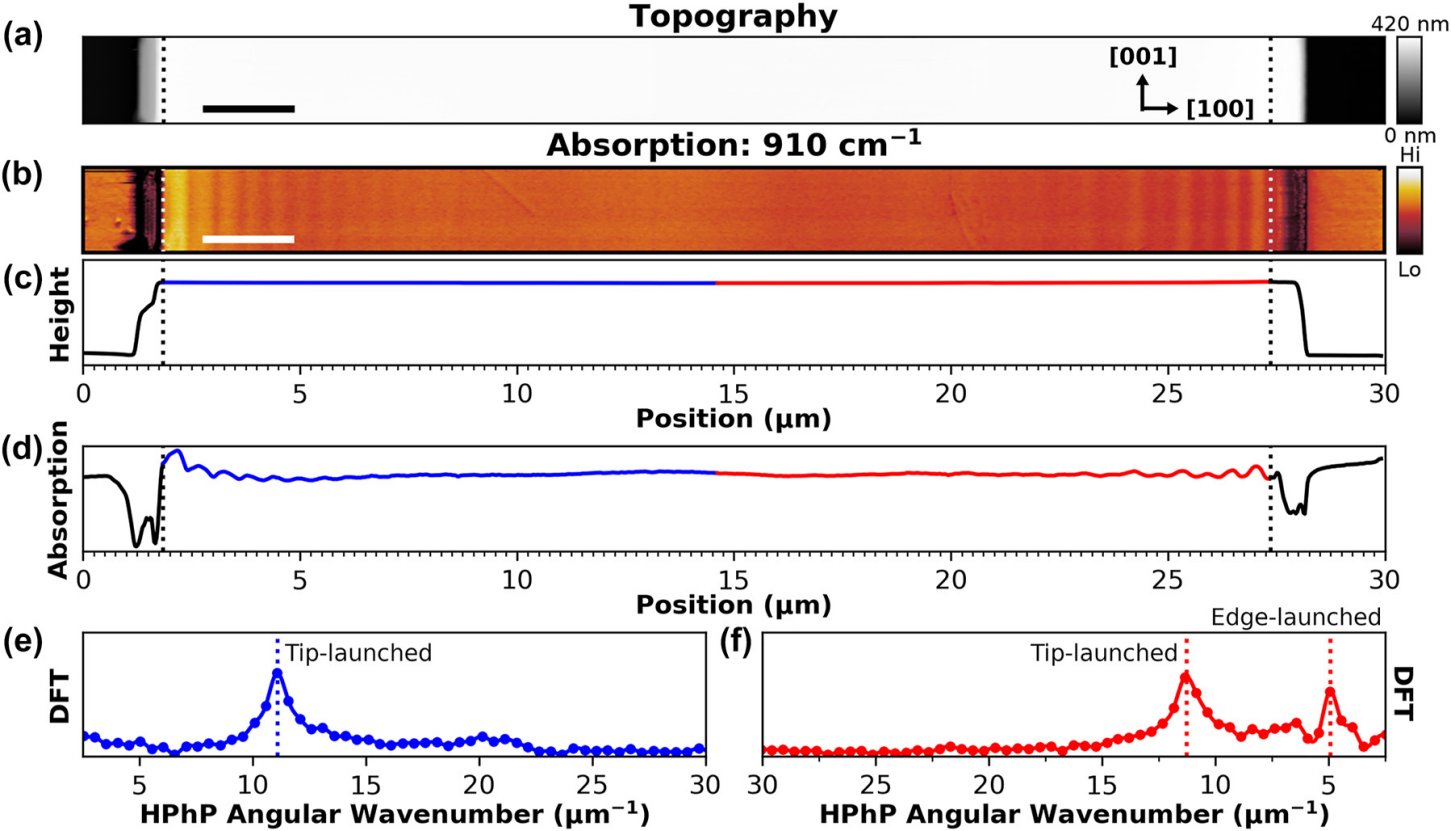
Real-space photothermal induced resonance (PTIR) absorption maps of hyperbolic phonon polaritons (HPhPs) propagating in a ^92^MoO_3_ flake (≈410 nm thick) deposited on an ultra-flat gold-coated SiO_2_ substrate. (a–b) Simultaneously acquired topography (a) and PTIR absorption (b) maps obtained with 910 cm^−1^ illumination. Scale bars represent 2 μm. (c–d) Average height (c) and absorption (d) profiles from (a) and (b), respectively, scaled to arbitrary units. The vertical dashed lines in (a–d) denote the positions of the topographically identified flake edges. The profiles have been split in half to analyze separately HPhPs launched or reflected from the edge opposite (left side, blue) and proximal (right side, red) to the incident illumination direction. (e–f) Discrete Fourier transforms (DFTs) of the measured absorption profile for the left (e) and right (f) edges of the α-MoO_3_ flake with the tip- and edge-launched HPhP modes labeled, scaled to arbitrary units. Only tip-launched HPhPs are observed at the flake edge opposite the illumination direction (left side), enabling a somewhat simplified analysis.

Since polaritons can be launched both by intrinsic features, such as a crystal edge, as well as by extrinsic features, such as an AFM tip, PTIR can detect both edge-launched and tip-launched HPhP modes within a single absorption map. These modes can be deconvolved by modeling the measured polaritons as the sum of multiple damped harmonic oscillations [78]. Significantly, the relative efficiencies for coupling light into edge-launched and tip-launched HPhPs have been found to depend upon the relative orientation between the crystal and the illumination angle [15], [67], [73], [79]. As such, to facilitate comparisons between different flakes, a rotation stage was used to align each flake such that the [001] direction (long edge) was perpendicular to the incident illumination direction. Since the Type II HPhPs observed here propagate from both [001] edges along the [100] direction in the plane of the flake, we observed fringes parallel to the [001] flake edges in PTIR maps (Figure 2). Furthermore, since the AFM fast scan direction (horizontal) is aligned perpendicular to the [001] edges, sequential line scans can be averaged along the slow scan direction (vertical), to yield one-dimensional profiles with improved signal-to-noise ratios (Figure 2d). The averaged one-dimensional real-space absorption profiles can be fit with Equation (1).

(1) $$\text{Absorption}\propto \underset{i}{\sum }A_{i}e^{\gamma_{i}x}\cos\left(k_{i}x+\varphi_{i}\right)$$

Here, *A* scales the model amplitude to match the observed intensity, *γ* is the decay or damping coefficient, *x* is the distance along the propagation direction, *k* is the angular wavenumber, and a phase parameter (*φ*) accounts for offsets in the relative positions of the measurement and model. Since PTIR absorption profiles consist of the superpositions of HPhPs, this model includes *i* independent modes, which are defined by peaks (*k*_*i*_) in discrete Fourier transform (DFT) power spectra.

As seen in Figure 2e and f, DFTs of the real-space PTIR profile reveal multiple peaks in frequency space. Notably a peak at ≈11.2 μm^−1^ is observed in the DFT power spectra calculated from the absorption profile near each edge, while a second peak at ≈5.0 μm^−1^ is observed for the right edge only (leading edge relative to the incident illumination). Based on the approximate doubling of the angular wavenumber between the two observed DFT peaks (≈5.0 μm^−1^ and ≈11.2 μm^−1^), and subsequent comparisons with calculated dispersion relationships, the lower frequency peak is assigned to an edge-launched mode, while the higher frequency mode observed for both edges is assigned to a tip-launched mode [14], [15], [33], [79].

Although edge-launched and tip-launched HPhP modes are manifestations of the same phenomenon, they appear as different peaks in the reciprocal-space power spectra due to their distinct detection mechanisms. An edge-launched HPhP originates from a crystal edge and is detected at the tip after propagating through the material. By contrast, a tip-launched HPhP originates at the probe tip position, propagates through the material, and is detected at the tip position only after reflecting back from the flake edge or other discontinuity. Therefore, for a given measurement position, due to the roundtrip, a tip-launched polariton will have traveled twice the distance (and will be more strongly attenuated) compared to polaritons launched by the crystal edge [79]. The relative coupling efficiency of the tip-launched mode compared to the edge-launched mode was found to be maximum at the edge opposite to the incident illumination (left side of Figure 2e). Therefore, this configuration was used to measure each flake, facilitating comparisons between measurements, maximizing signal-to-noise ratios, and simplifying the data analysis to the consideration of HPhPs as a single tip-launched mode. As an added benefit, the quality of the flake edge was found to have less of an effect on the intensity of the tip-launched HPhP modes in PTIR maps. The excitation efficiency (i.e., relative intensity) of edge-launched modes was found to be comparatively more sensitive to the edge quality. As a result, we fit the real-space absorption profiles using Equation (2) [80].

(2) $$\text{Absorption}\propto \frac{1}{\sqrt{x}}Ae^{-2x\gamma }\cos\left(2xk+\varphi \right)$$

Here, the same parameters are used as in Equation (1) but a $1/\sqrt{x}$ prefactor scales the intensity of the tip-launched mode, which spreads from a point source, compared to an edge-launched mode that propagates as a plane wave [79]. This method has been previously established to provide an accurate approximation [33]. Additionally, the roundtrip distance of a tip-launched mode is represented by 2*x*, where *x* is the distance from the flake edge in PTIR absorption profiles. The details and an example of this fit are included in the Supplementary Material as Figure S2.

Several characteristics of HPhPs can be obtained from the parameters of the model fit to the PTIR absorption profiles. The propagation lengths (*L*_P_) of HPhPs are related to the harmonic damping coefficient according to Equation (3).

(3) $$L_{\text{P}}=\frac{1}{\gamma }$$

The group velocities (*ν*_g_) of HPhPs through the α-MoO_3_ flake can be derived from the slope of the dispersion curve,

(4) $$\nu_{\text{g}}=\frac{\partial \omega }{\partial k}$$

Finally, the lifetimes of HPhPs can be approximated as:

(5) $$\tau \approx \frac{L_{\text{P}}}{\nu_{\text{g}}}$$

Altogether, this method and analysis provides a useful tool to extract the dispersion and characteristics of HPhPs from real-space near-field images.

**Figure 3:**
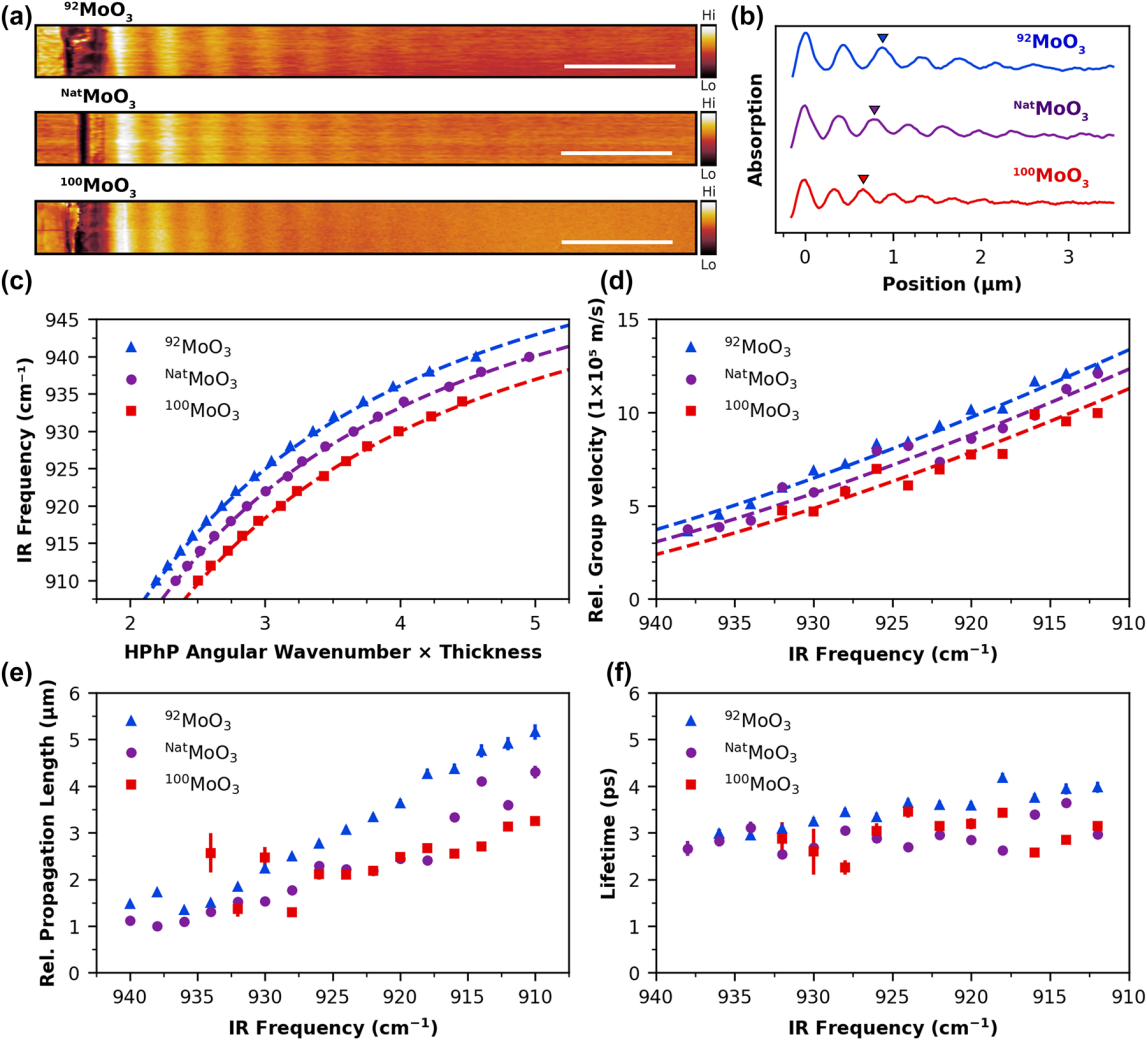
Photothermal induced resonance (PTIR) absorption maps of hyperbolic phonon polaritons (HPhPs) and their analysis in natural and isotope-enriched α-MoO_3_ crystals. (a) PTIR absorption maps at 910 cm^−1^ for a ^92^MoO_3_ flake (≈307 nm thick; top), ^Nat^MoO_3_ flake (≈295 nm thick; middle) and ^100^MoO_3_ flake (≈270 nm thick; bottom). Scale bars are 1 μm. (b) Average absorption profiles for the maps shown in (a). The profiles are aligned based on the position of the first maximum. The position of the third maxima in each profile is indicated with a triangle to highlight the effects of isotopic enrichment. (c–f) Comparison of (c) HPhP dispersion relations, (d) group velocities, (e) propagation lengths and (f) lifetimes for α-MoO_3_ flakes of similar thicknesses and different isotopic composition. The dashed lines in c and d represent the theoretical HPhP dispersion (see Equation (6)) with the dielectric fitting parameters tuned for each isotopic composition (see Table 1 for the values used) and the derivative of the theoretical dispersion (*n* = 1) for the natural and isotope-enriched α-MoO_3_ flakes, respectively. In the plots of relative group velocities (d) and relative propagation lengths (e), the measurements corresponding to isotopically enriched MoO_3_ have been normalized relative to ^Nat^MoO_3_ based on their measured thicknesses. Error bars in (c–f) represent uncertainties propagated from least-squares fit covariance matrices.

To understand the effects of isotopic enrichment, PTIR maps of the Type II HPhPs were measured in the spectral range between ≈910 cm^−1^ and 945 cm^−1^ for naturally abundant (^Nat^MoO_3_) and isotopically enriched (^92^MoO_3_ and ^100^MoO_3_) single crystals of similar thickness (≈300 nm), see Figure 3a. At first glance, the effects of isotopic enrichment are not immediately apparent in PTIR absorption maps and the extracted profiles, as the decaying oscillations seem quite similar (Figure 3b). However, compared to the dispersion relationship in ^Nat^MoO_3_, on average the dispersions in ^92^MoO_3_ and ^100^MoO_3_ are clearly blue- (≈−7 %) and red-shifted (≈+9 %), respectively (Figure 3c). To elucidate the effects of isotopic enrichment on the dispersion of HPhPs in MoO_3_, as well as control for the effects of flake thickness [81], an analytic function specific to the in-plane HPhPs that propagate along the [100] direction, Equation (6) [82], [83], was fit to the PTIR-measured dispersion curves.

The optical phonon modes (*ω*_TO_ and *ω*_LO_) were constrained based on previously published spectroscopic measurements [35] and allowed to vary within reason (Table S1) to fit Equation (6) to the measurements. While HPhP propagation critically depends on the flake thickness [84], this method yields a dimensionless quantity, here defined as HPhP angular wavenumber × thickness, which enables the comparison of ^92^MoO_3_, ^Nat^MoO_3_, and ^100^MoO_3_ of slightly different thicknesses.

(6) $$\begin{array}{ll} \tilde{k}\left(\omega \right) & =\left(k+i\kappa \right)d\\ & =-\psi \left[\arctan\left(\frac{\varepsilon_{\text{a}}}{\varepsilon_{\left[100\right]}\psi }\right)+\arctan\left(\frac{\varepsilon_{\text{s}}}{\varepsilon_{\left[100\right]}\psi }\right)+n\pi \right] \end{array}$$

$$\text{for }\psi =\frac{\sqrt{\varepsilon_{\left[010\right]}}}{i\sqrt{\varepsilon_{\left[100\right]}}}.$$

Here, the complex HPhP wavenumber ( $\tilde{k}$ ) depends on the frequency (*ω*) of the incident IR light, the thickness (*d*) of the α-MoO_3_ flake, and the frequency-dependent permittivities, *ɛ*_[100]_ and *ɛ*_[010]_ of α-MoO_3_, along the respective crystallographic directions as defined in Figure S1. *ɛ*_a_ represents the permittivity of air and *ɛ*_s_ represents the frequency-dependent permittivity of the substrate, gold in this case. Finally, *n* denotes the mode order. The real component of the HPhP wavenumber (*k*) can be obtained through the spatial frequency of the fringes observed in real-space PTIR and s-SNOM images, while the imaginary component (*κ*) is related to the polariton damping.

This method yields the parameters defining the reststrahlen bands as well as provides a means to understand the dispersion and properties of HPhPs. Specifically, we obtain the relative electric permittivities at the high-frequency limit, and the frequencies of the TO and LO phonon modes delimiting the reststrahlen bands for both the naturally abundant and isotopically enriched α-MoO_3_ flakes. The values obtained through this process are reported in Table 1 and are compared with previously published values in Table S1 of the Supplementary Material.

**Table 1:** Experimentally determined parameters for the investigated reststrahlen band in both isotopically enriched and natural α-MoO_3_.

	[100]				[010]			
	*ε*_∞_	*ω*_TO_ (cm^−1^)	*ω*_LO_ (cm^−1^)	Γ (cm^−1^)	*ε*_∞_	*ω*_TO_ (cm^−1^)	*ω*_LO_ (cm^−1^)	Γ (cm^−1^)
^92^MoO_3_	3.7	822	976	3.7	2.3	963	1008	0.8
^Nat^MoO_3_	4.0	819	975	4	2.6	960	1006	2
^100^MoO_3_	4.2	816	974	3.8	3.0	956	1002.5	0.8

Since the thickness of the crystals can have significant effects on the dispersion of HPhPs and their group velocities, propagation lengths [82], in Figure 3 we compare flakes with similar thickness (≈300 nm), see also Figure S3a for the respective AFM topographs. Substantially thicker (≈500 nm, Figure S3b) flakes were also measured and compared in Figure S4. The group velocities and propagation lengths were found to linearly depend on the thickness of the hyperbolic material within the thickness ranges evaluated here (Equation (6)) [82]. As a result, their values have been normalized to account for small differences in thickness compared to the ^Nat^MoO_3_ flake. This was performed by multiplying the measured value by *d*_Nat_/*d*_Iso_, where *d*_Nat_ is the thickness of the ^Nat^MoO_3_ flake, and *d*_Iso_ is the thickness of the ^92^MoO_3_ or ^100^MoO_3_ flake. We experimentally validated this approach by comparing flakes of similar isotopic composition but significantly different thicknesses (≈300 nm vs. ≈500 nm) (Figure S5). By contrast, since the lifetimes are obtained as ratios of the propagation length and group velocity, which are both dependent on thickness, no normalization for thickness was found to be necessary (Figure 3f).

Notably, we first fit the analytic function to the data sets obtained for ≈300 nm thick flakes to obtain the defining parameters. Following that, we used these same parameters to fit the dispersion of HPhPs in the ≈500 nm thick flakes (Figure S4), with excellent agreement. In this way we obtained the relative electric permittivities at the high frequency limit (*ε*_∞_), and the frequencies of the TO and LO phonon modes (*ω*_TO_ and *ω*_LO_) corresponding to the reststrahlen bands of the three isotopic compositions along with their respective linewidths (Γ).

Subsequently, we used the analytic function Equation (6) to obtain thickness dependent HPhP angular wavenumbers (μm^−1^) compared to the previously described unitless HPhP angular wavenumber × thickness. Together with the experimentally derived parameters (Table 1) we visualized the effects of isotopic enrichment in theoretical flakes of identical thickness (e.g., exactly 300 nm and 500 nm, Figure S6), corroborating our observations of altered dispersions in a manner that is independent of thickness. Plots of the analytic model in terms of HPhP angular wavenumber × thickness are also included in Figure S6 for completeness. Isotopic enrichment also affects the group velocities obtained via Equation (4). Compared to ^Nat^MoO_3_, the lighter isotope, ^92^Mo, results in a faster group velocity (≈+12 %) in ^92^MoO_3_, while the heavier isotope, ^100^Mo, results in a slower (≈−12 %) group velocity in ^100^MoO_3_ with respect to incident IR frequency. The derivatives (group velocities) of the optimized analytic functions also show a similar trend in good agreement with the experimentally determined values (Figure 3d). In contrast to previous studies that reported significant improvements to the propagation lengths and lifetimes of elliptical PhPs [15], [29], [35], for example up to a ≈70 % improvement reported in ^92^MoO_3_ [35], here we observe only a slight improvement (≈20 %) of the lifetimes of HPhPs in ^92^MoO_3_. We also evaluated the effects of isotopic enrichment with a figure of merit for flakes of both sets of thicknesses (Figure S7), which again showed slight improvements over ^Nat^MoO_3_. We note that the HPhP lifetimes in ^Nat^MoO_3_ previously reported in one of our papers [14], approximately twice as long as the lifetimes reported in Figure 3f, were overestimated by a factor of 2.

**Figure 4:**
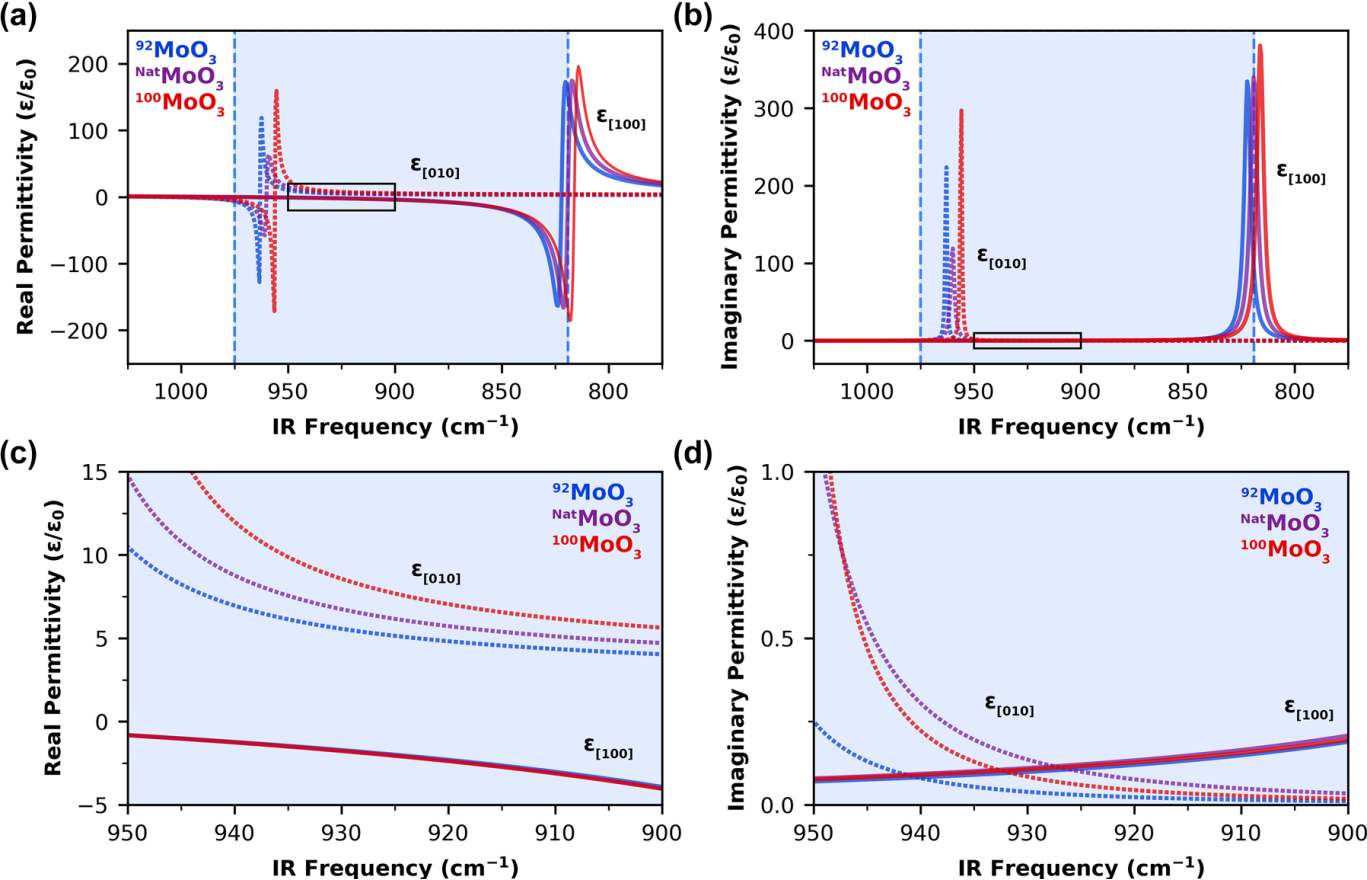
Calculated real (a) and imaginary (b) components of the complex dielectric function (*ε*) of naturally occurring and isotope-enriched α-MoO_3_ relative to that of free-space (*ε*_0_) along the [100] and [010] directions. The blue background delimits the in-plane HPhPs (Type II) reststrahlen band of α-MoO_3_. (c–d) Magnified views of the spectral region where the PTIR measurements were obtained for both real (c) and imaginary (d) components; also highlighted with black rectangles in (a–b).

The values derived from the analysis of real-space PTIR images were used to plot the real components of the complex dielectric function using the Lorentz oscillator permittivity model, Equation (S1), for naturally abundant and isotope-enriched α-MoO_3_. As shown in Figure 4, isotopic enrichment clearly shifts the optical phonon frequencies and, therefore, shifts the real and imaginary permittivity curves. Additionally isotopic enrichment can also reduce the imaginary permittivity values by reducing isotopic disorder and associated optical losses (Figure 4b and d). We note that isotopic enrichment in α-MoO_3_ induces significantly smaller spectral shifts (≈3.5 cm^−1^, ≈0.4 %) than in hBN (up to 27 cm^−1^, ≈2 %) [29] due to the smaller relative change in the isotope mass. In the spectral range analyzed in this work (910 cm^−1^ to 940 cm^−1^), the real part of the in-plane permittivity Re(*ε*_[100]_) is practically coincident for the three isotopically distinct samples (Figure 4a and c). Therefore, the comparison of the polariton properties in Figure 3 and Figure S4, as a function of the IR frequency, also intrinsically provides a comparison near parity of the real in-plane [100] permittivity. Hence, the modest improvements in propagation lengths (Figure 3e) and lifetimes (Figure 3f) can be attributed to a reduction of scattering due to isotopic disorder, in agreement with the imaginary permittivity plots (Figure 4b and d).

**Figure 5:**
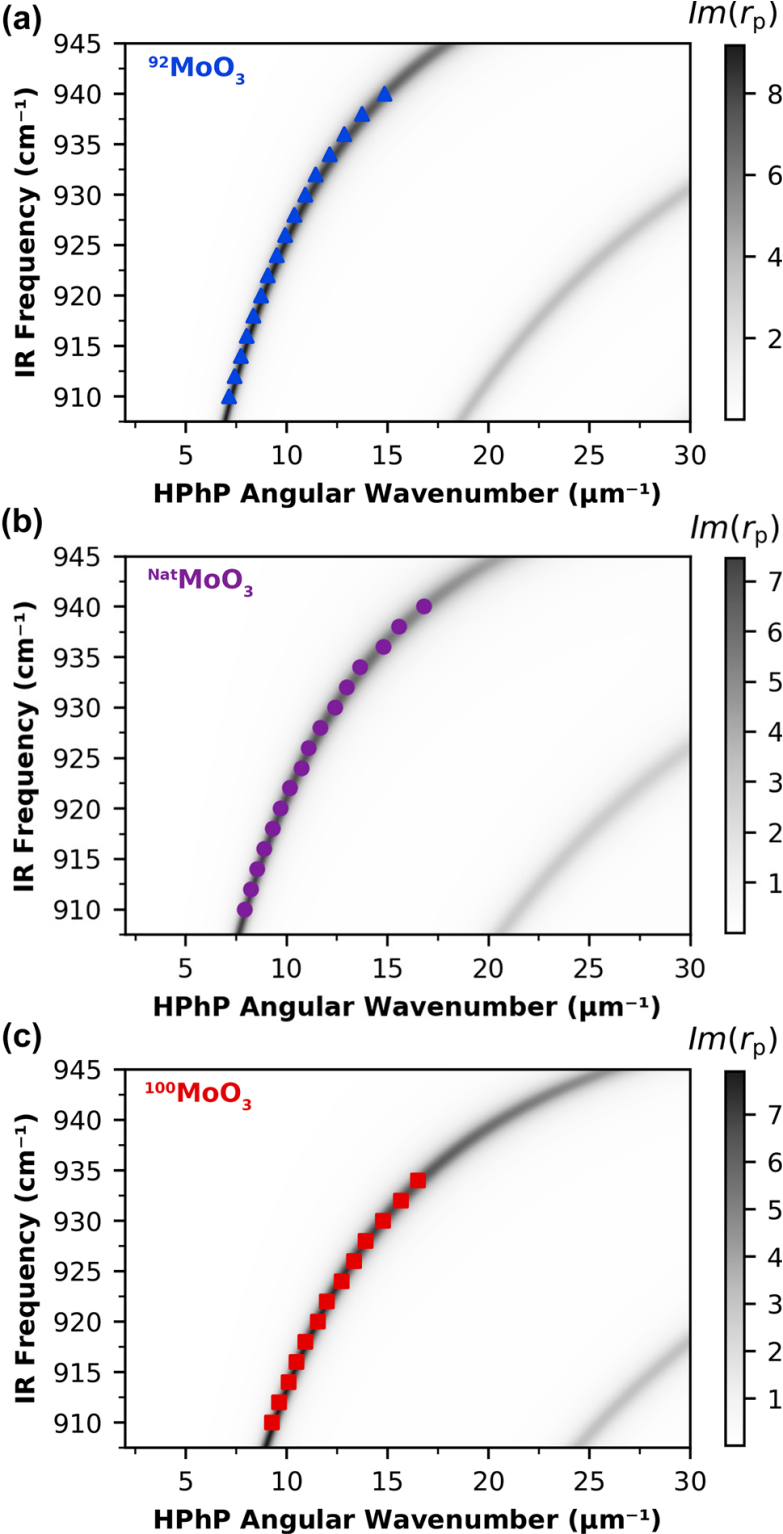
Comparisons of measured in-plane hyperbolic phonon polariton (HPhP) dispersions (solid markers) to the imaginary component, $Im\left(r_{\text{p}}\right)$ , of the complex Fresnel reflectance of a three-layer model system (air/MoO_3_/gold substrate). Results are presented for the ^92^MoO_3_ (a), ^Nat^MoO_3_ (b), and ^100^MoO_3_ (c) flakes characterized in Figure 3.

Finally, the dispersion of HPhPs can also be estimated by calculating the complex Fresnel reflectances of the three-layer (air/MoO_3_/substrate) system, as described in the Supplementary Material by Equation (S5) [85]. Figure 5 compares the calculated HPhP angular momenta of different orders (dark stripes) with the PTIR measured values (solid points). In all cases, the calculated principal branch corresponding to the fundamental mode (*n* = 1) agrees well with the experimental data. Similarly good agreement was found for the thicker (≈500 nm) flakes (Figure S8), further validating the parameters reported in Table 1.

## 3 Conclusions

PTIR absorption maps were used to visualize in real space and characterize the fundamental parameters of HPhPs propagating in natural and isotope-enriched α-MoO_3_ single crystals. By maintaining consistent measurement conditions, comparing α-MoO_3_ crystals with similar thickness, as well as normalizing the properties of HPhPs relative to flake thickness, we isolated the effects of isotopic enrichment on the characteristics and optical properties of HPhPs. We found that ^92^MoO_3_ and ^100^MoO_3_ isotopic enrichment permits tuning (i.e., shifting) the α-MoO_3_ optical dispersion (angular wavenumber × thickness vs. IR frequency) by ≈−7 % and ≈+9 %, respectively. Similarly, the group velocities and propagation lengths were mildly affected by isotopic enrichment, with the lifetimes showing only slight improvements compared to ^Nat^MoO_3_ for the in-plane HPhPs measured here. Isotope engineering was found to be a viable strategy to tune the dispersion of HPhPs, potentially offering a useful method to finely control the in-plane anisotropic propagation of HPhPs in α-MoO_3_ towards nanophotonic applications while preserving the lifetimes of the naturally abundant material.

## 4 Methods

### 4.1 Synthesis of single-crystalline α-MoO_3_

α-MoO_3_ flakes were grown by the physical vapor transport method using MoO_3_ powders containing either naturally abundant Mo (Alfa Aesar, 99.9995 %) or isotopically enriched ^92^Mo and ^100^Mo (Isoflex USA, enrichment level 99.93 % and 99.01 %, respectively). The powders were loaded in quartz ampoules that were sealed under vacuum and placed in a 3-zone furnace. The MoO_3_ powder charge was kept at 800 °C, whereas the large single-crystalline α-MoO_3_ belts were formed over 8 h along the ≈10 cm of the ampoule length sitting at 750 °C. For the measurements, α-MoO_3_ flakes were exfoliated onto Au-coated glass substrates, a commonly used substrate for PTIR analyses [14], [57], where the measurement benefits from the ultraflat surface.

### 4.2 Photothermal induced resonance measurements

#### 4.2.1 Phonon polariton imaging

All PTIR measurements were acquired using the resonance enhanced excitation scheme [51] with commercially available gold-coated Si contact mode AFM probes (nominal spring constant of 0.07 N/m–0.4 N/m and a first resonance frequency in air of 13 kHz ± 4 kHz). A quantum cascade laser (QCL) array with a tunable pulse repetition rate (1 kHz to 2000 kHz) and IR frequency (910 cm^−1^ to 1905 cm^−1^) illuminated a ≈50 μm diameter region of the sample around the probe tip. Laser light (p-polarized) was obliquely incident (20° from the sample surface) and aligned perpendicular to the [001] axis of the α-MoO_3_ flakes in the in-plane direction. This illumination geometry was maintained across all samples to avoid potential disparities in HPhP launching efficiencies. Further, to avoid the detection of edge-launched modes, that may complicate analysis, the trailing edge of all α-MoO_3_ was considered exclusively to ensure that tip-launched HPhPs were predominant across all analyzed datasets. A phase-locked loop with a bandwidth of 50 kHz to 100 kHz was used to maintain resonant excitation by adjusting the repetition rate of the laser pulses to match one of the contact-resonance modes of the cantilever (≈400 kHz), leading to a *Q*/2*π* amplification of the PTIR signal, where *Q* is the quality factor of the cantilever oscillation [45]. Absorption maps were acquired by raster scanning the probe while the sample was illuminated with a constant incident IR frequency.

#### 4.2.2 Image processing and analysis

Topographic and PTIR absorption map pairs acquired simultaneously were analyzed to estimate the HPhPs properties as described in detail elsewhere [14]. In brief, the edges of α-MoO_3_ flakes were first identified from plane-subtracted AFM topographs by applying a Canny edge detection algorithm. This procedure separates images into regions inside and outside the measured flakes. The 2D AFM and PTIR maps were averaged in the vertical direction and only regions inside the flake were used for the subsequent analysis and processing. Exponential decaying lines of best fit and arithmetic means were subtracted from the averaged PTIR absorption profiles to obtain corrected absorption profiles. Discrete Fourier transforms of the corrected absorption profiles were used to identify the frequency of HPhP modes within the α-MoO_3_ crystals based on the appearance of peaks in the power spectra in Fourier space. The characteristics of the detected HPhPs were defined by fitting the model described in Equation (2) to filtered absorption profiles beginning from the initial angular wavenumber identified in power spectra. Absorption signals were high-pass filtered with an exponential damping function with a cutoff around 65 % of the first detected peak position to isolate the frequency of the HPhP without attenuating it for analysis (Figure S2). Group velocities were approximated by calculating the numerical derivative of the HPhP dispersion relationship for both the measured data and analytic function by using the symmetric finite difference method. Uncertainties reported for all quantities represent one standard deviation in the mean value. For quantities derived from least-square fits, the values reported derive from the propagation of uncertainties obtained from the covariance matrices. Since the optimized fitting parameters were determined for each PTIR absorption profile independently, the reported error bars only estimate the fitting uncertainties for a single image and do not account for variability between images.

The parameters that define the frequency-dependent electric permittivity of α-MoO_3_ were obtained through optimizing the fit of the analytic function (Equation (6)), with consideration of previously reported shifts observed in spectroscopy of isotopically enriched α-MoO_3_ [35], and the imaginary component of the complex Fresnel reflectance (Equation (S5)) for one set of flakes with a similar thickness (Figure 2). These values were then validated for a separate set of thicker flakes (Figure S4) and appear in Table 1 and are compared with previously published values in Table S1.

## Supplementary Material

Supplementary Material Details
