# Supplementary material for: Isotopic effects on in-plane hyperbolic phonon polaritons in MoO3
Source: Nanophotonics. 2024 Mar 4;13(9):1581–92. doi: 10.1515/nanoph-2023-0717 (PMC11155493; doi:10.1515/nanoph-2023-0717)
Supplement: Supplementary file 1 — Supplementary Material Details [file j_nanoph-2023-0717_suppl_001.docx]

***Supplementary Material:***

**Isotopic effects on in-plane hyperbolic phonon polaritons in MoO_3_**

*Jeremy F. Schultz,^1^ Sergiy Krylyuk,^2^ Jeffrey J. Schwartz,^1,3^ Albert V. Davydov,^2^ and Andrea Centrone^1^*

*^1^*Physical Measurement Laboratory, National Institute of Standards and Technology, Gaithersburg, Maryland 20899, United States of America

^2^Material Measurement Laboratory, National Institute of Standards and Technology, Gaithersburg, Maryland 20899, United States of America

*^3^*Department of Electrical and Computer Engineering, University of Maryland, College Park, Maryland 20742, United States of America

**S1 Structural and Spectroscopic Properties of α‑MoO_3_**

**S1.1 Lorentz Oscillator Permittivity Model**

The frequency-dependent electric permittivities of natural and isotope-enriched α-MoO_3_ (shown in **Figures 1 and 5**) were computed using the Lorentz oscillator model described by **Equation S1**.

| $\varepsilon_{j}\left( \omega\right)= \varepsilon_{\infty,j}\left( 1+\frac{\omega_{LO,j}^{2}-\omega_{TO,j}^{2}}{\omega_{TO,j}^{2}- \omega^{2}-i\omega\Gamma} \right)$ | (S1) |
| --- | --- |
| for $j=\left[ 100 \right], [010]$ |  |

In this model, $\varepsilon_{\infty}$ is the permittivity in the high frequency limit (as ω 🡪 ∞), ω_TO_ and ω_LO_ are the transverse optical (TO) and longitudinal optical (LO) phonon modes, *Γ* is the phonon damping term, and the subscripts denote the crystallographic directions relevant to the propagation of Type II hyperbolic phonon polaritons (HPhPs) that occur within the spectral region of the investigated reststrahlen band. [100] is the in-plane direction of propagation of HPhPs, while [010] is the crystal stacking direction. Previously reported values for these parameters for both natural and isotope-enriched α-MoO_3_ are summarized in **Table S1** along with the values determined in this work from the analysis of real-space imaging of HPhPs with photothermal induced resonance (PTIR) maps.


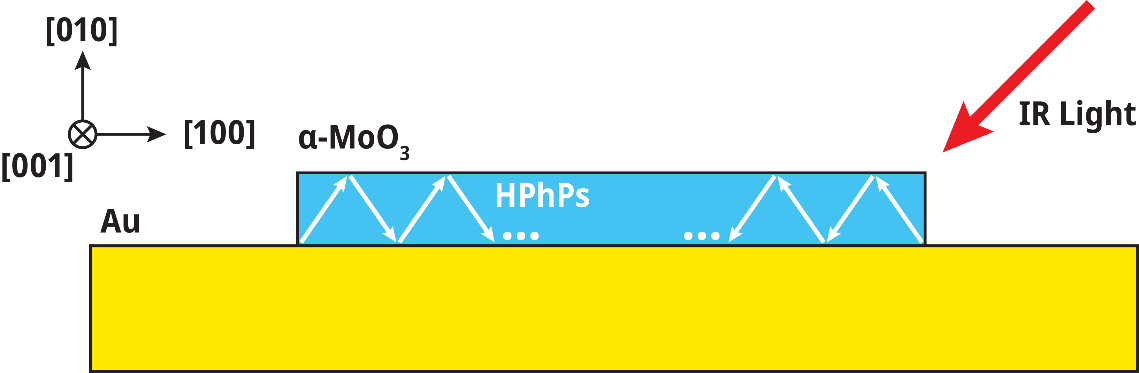


**Figure S1:** Scheme illustrating the propagation of edge-launched hyperbolic phonon polaritons (HPhPs) in α-MoO_3_ in the air/α-MoO_3_/Au layered structure. Type II HPhPs measured in this work propagate within the plane of incidence along the [100] direction as shown in this cross-sectional view normal to the [001] axis.

**Table S1:** Summary of parameters that define the reststrahlen band examined in this work for both natural and isotope-enriched α-MoO_3_.

| **Material** | **Source** | **Parameters*^a^*** | | | | | | | |
| --- | --- | --- | --- | --- | --- | --- | --- | --- | --- |
|  |  | **[100]** | | | | **[010]** | | | |
|  |  | ***ε*_∞_** | ***ω*_TO_**  (cm^-1^) | ***ω*_LO_** (cm^-1^) | ***Γ***  (cm^-1^) | ***ε*_∞_** | ***ω*_TO_**  (cm^-1^) | ***ω*_LO_** (cm^-1^) | ***Γ***  (cm^-1^) |
| ^92^MoO_3_ | Ref. [S1] | 4.7 | 820 | 978 | 3.7 | 2.6 | 964 | 1008 | 0.8 |
| ^92^MoO_3_ | This Work | 3.7 | 822 | 976 | 3.7 | 2.3 | 963 | 1008 | 0.8 |
| ^Nat^MoO_3_ | Ref. [S2] | 4.0 | 820 | 972 | 4 | 2.4 | 958 | 1004 | 2 |
| ^Nat^MoO_3_ | Ref. [S3] | 5.78 | 821.4 | 963.0 | 6.0 | 4.47 | 956.7 | 1006.9 | 1.5 |
| ^Nat^MoO_3_ | Ref. [S4] | — | 820 | 963 | — | — | 963 | 1003 | — |
| ^Nat^MoO_3_ | Ref. [S5] | — | 825 | 975 | — | — | 967 | 1008 | — |
| ^Nat^MoO_3_ | Ref. [S6] | — | 818 | 974 | — | — | 962 | 1010 | — |
| ^Nat^MoO_3_ | Ref. [S1] | 5.0 | 818 | 976 | 4.0 | 2.0 | 960.5 | 1006 | 1.5 |
| ^Nat^MoO_3_ | This Work | 4.0 | 819 | 975 | 4 | 2.6 | 960 | 1006 | 2 |
| ^100^MoO_3_ | Ref. [S1] | 4.2 | 816 | 975 | 3.8 | 3.0 | 956 | 1002.5 | 0.8 |
| ^100^MoO_3_ | This Work | 4.2 | 816 | 974 | 3.8 | 3.0 | 956 | 1002.5 | 0.8 |

*^a^*Relative electric permittivity at the high-frequency limit (*ε*_∞_), transverse optical (TO) and longitudinal optical (LO) phonon mode frequencies (*ω*), and line widths (*Γ*) of α‑MoO_3_, for the crystallographic directions [100], the propagation direction of the hyperbolic phonon polaritons measured in this work and [010], the crystal stacking axis.

**S2 PTIR Absorption Profile Fitting**

PTIR absorption profiles were fit using the damped harmonic oscillator model described in the manuscript (see also 4.2.2 Image processing and analysis) according to:

| $\mathrm{Absorption}\propto\frac{1}{\sqrt{x}}Ae^{-2x\gamma}\cos\left( 2xk+\varphi\right)$ | (S2) |
| --- | --- |

**Figure S2** is an example of this fitting process where measured (black), filtered (blue), and fitted (red) absorption profiles at 910 cm^-1^ are shown in both real space and reciprocal (Fourier) space. A single prominent peak corresponding to a tip-launched mode was detected in the discrete Fourier transform (DFT) power spectrum, filtered, and then fitted in real space by optimizing the parameters: $A$, $\gamma$, $k$, and $\varphi$ in **Equation (S3)** to characterize the observed HPhP. The parameters were optimized using a Trust Region Reflective algorithm with the following constraints: $A\in\left[ 0, 10 \right]$, $\gamma\in\left[ 0.01, 4 \right]$ µm^‑1^, $\varphi\in\left[ -\pi, \pi\right]$, and $k$ maintained within narrow regions centered around the respective DFT peaks, $\left[ k_{peak}\pm\Delta\right]$, for $\Delta$ ≈ 3.0 µm^‑1^.


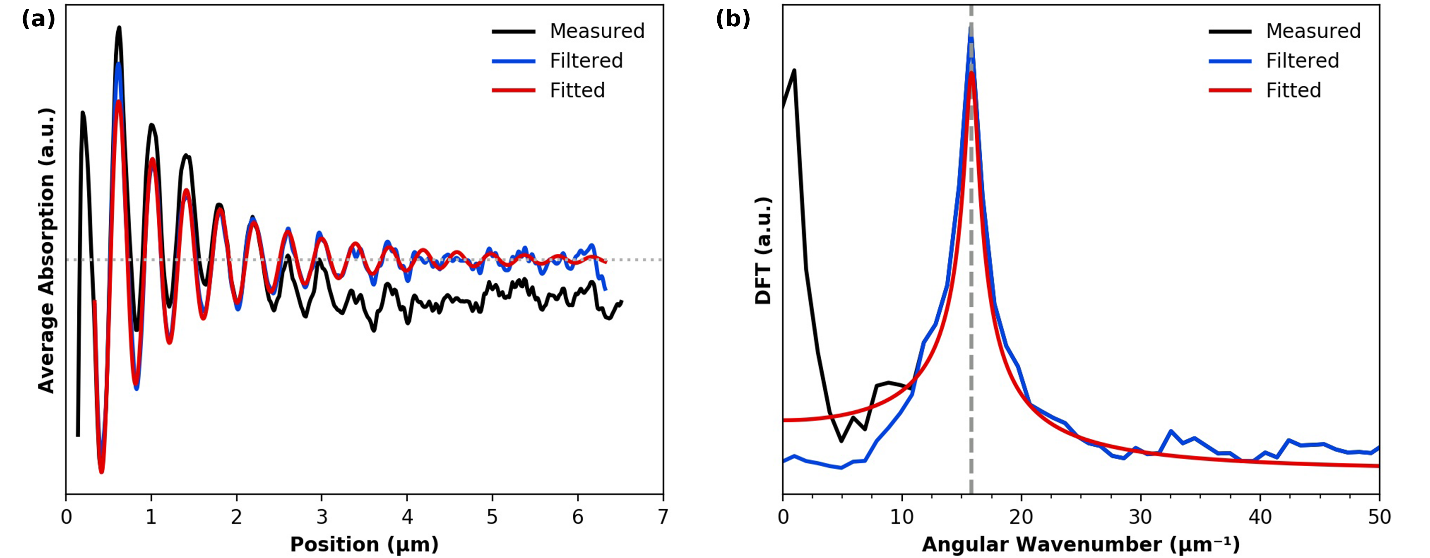


**Figure S2:** Example of the analysis of a PTIR absorption profile by fitting with a damped harmonic oscillator model used throughout this work. (a) Real-space fitting of the measured absorption profile for 910 cm^-1^ illumination of the ^92^MoO_3_ flake as shown in Figure 3(a). The measured (black), filtered (blue), and model-fit (red) absorption profiles are plotted simultaneously for comparison. (b) Discrete Fourier transforms (DFTs) of the measured (black), filtered (blue), and model-fit (red) absorption profiles plotted in (a). The vertical dashed gray line denotes the position of the observed hyperbolic phonon polariton in reciprocal space.

**S3 Measurements and Comparison of Hyperbolic Phonon Polaritons in Thicker Flakes**

**
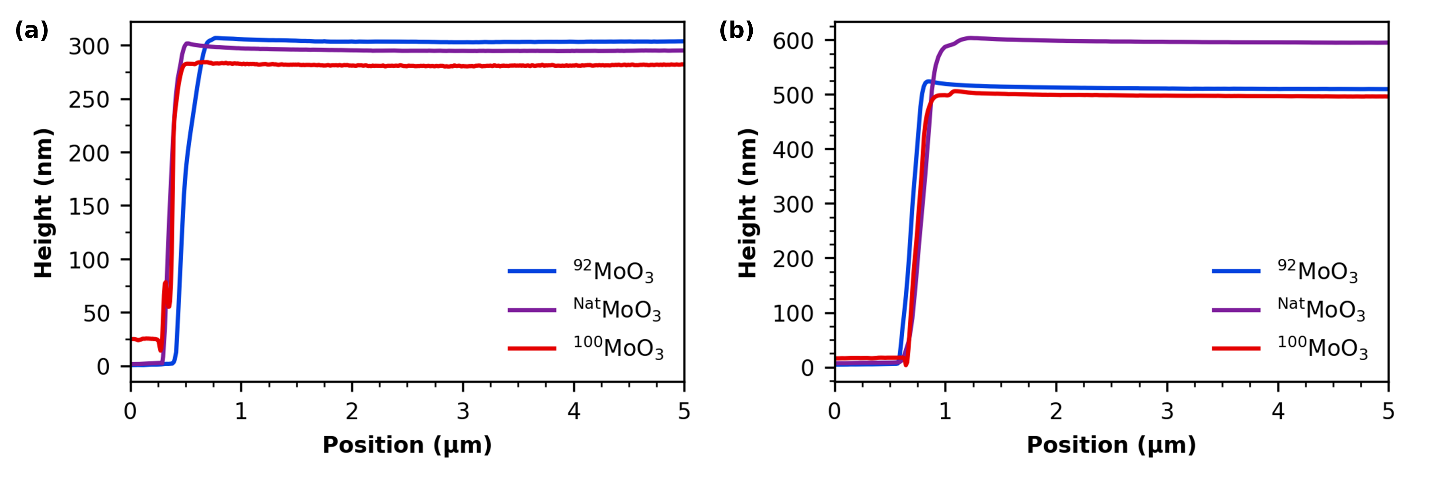
Figure S3:** Average height profiles of the natural and isotope enriched α-MoO_3_ flakes acquired simultaneously with the absorption maps. (a) Average height profiles of flakes measured for **Figures 3 and** **4**. (b) Average height profiles of flakes measured for **Figures S4**and**S6**.

**
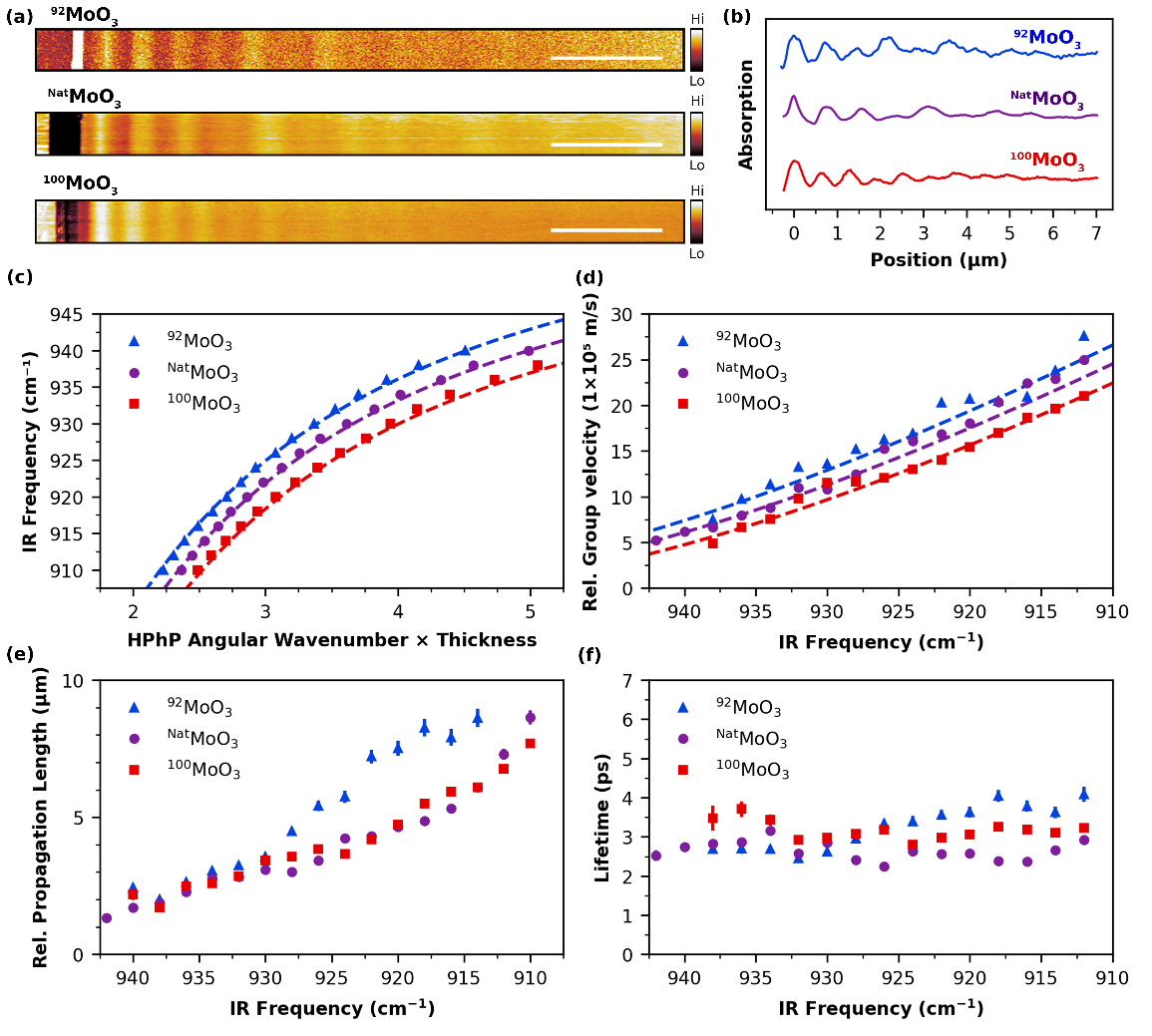
**

**Figure S4:** Real-space photothermal induced resonance (PTIR) measurements of hyperbolic phonon polaritons (HPhPs) propagation and analysis in natural and isotope enriched α-MoO_3_ slightly thicker than those considered in Figure 3. (a) PTIR absorption maps of at 910 cm^-1^ for a ^92^MoO_3_ flake (≈ 503 nm thick; top), ^Nat^MoO_3_ flake (≈ 587 nm thick; middle), and ^100^MoO_3_ flake (≈ 495 nm thick; bottom). Scale bars are 2 μm. (b) Average absorption line profiles for the maps shown in panel-a. (c-f) Comparison of (c) HPhP dispersion relations, (d) group velocities, (e) propagation lengths, and (f) lifetimes for α-MoO_3_ flakes of similar thicknesses and different isotopic composition. The dashed lines in c, d represent the theoretical HPhP dispersion (see **Equation 6**) with the parameters tuned (see **Table 1** for a table of the values used) and the derivative of the first-order (*n* = 1) theoretical dispersion for the respective natural and isotope-enriched α-MoO_3_ flakes. In the plots of relative group velocities (d) and relative propagation lengths (e), the measurements corresponding to isotopically enriched MoO_3_ have been normalized relative to ^Nat^MoO_3_ based on their measured thicknesses. Error bars in (c–f) represent uncertainties in the mean values, propagated from least-squares fit covariance matrices.
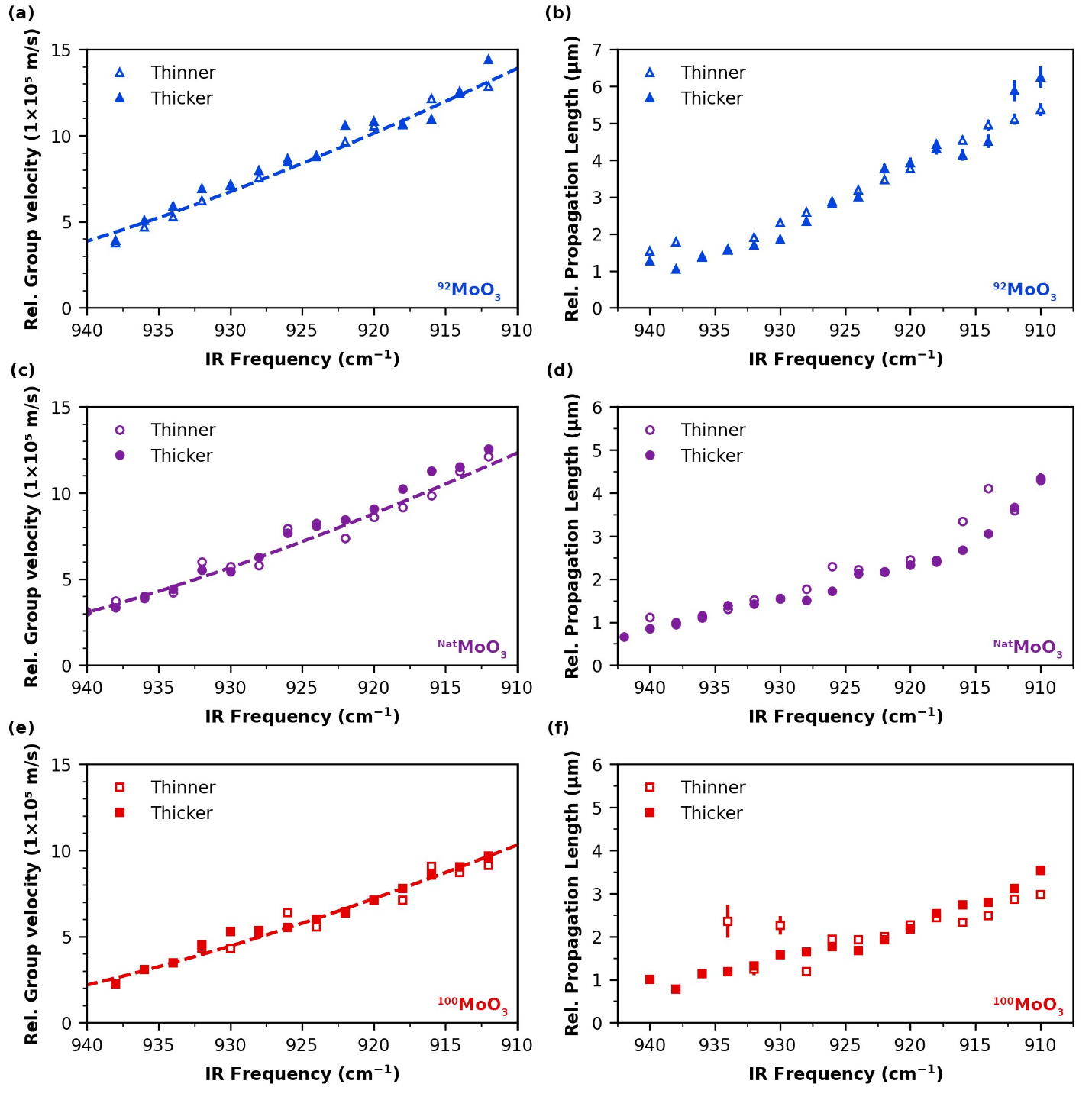


**Figure S5:** Comparison of the thickness-dependent properties of hyperbolic phonon polaritons (HPhPs), group velocity and propagation length, for natural and isotope-enriched α-MoO_3_, ^92^MoO_3_ (a,b), ^Nat^MoO_3_ (c,d), and ^100^MoO_3_ (e,f) for the two data sets of significantly different thicknesses (Thinner: ≈ 300 nm: Figure 3; Thicker: ≈ 500 nm: Figure S4). The data has been normalized for the thickness of the thinner flake.

**
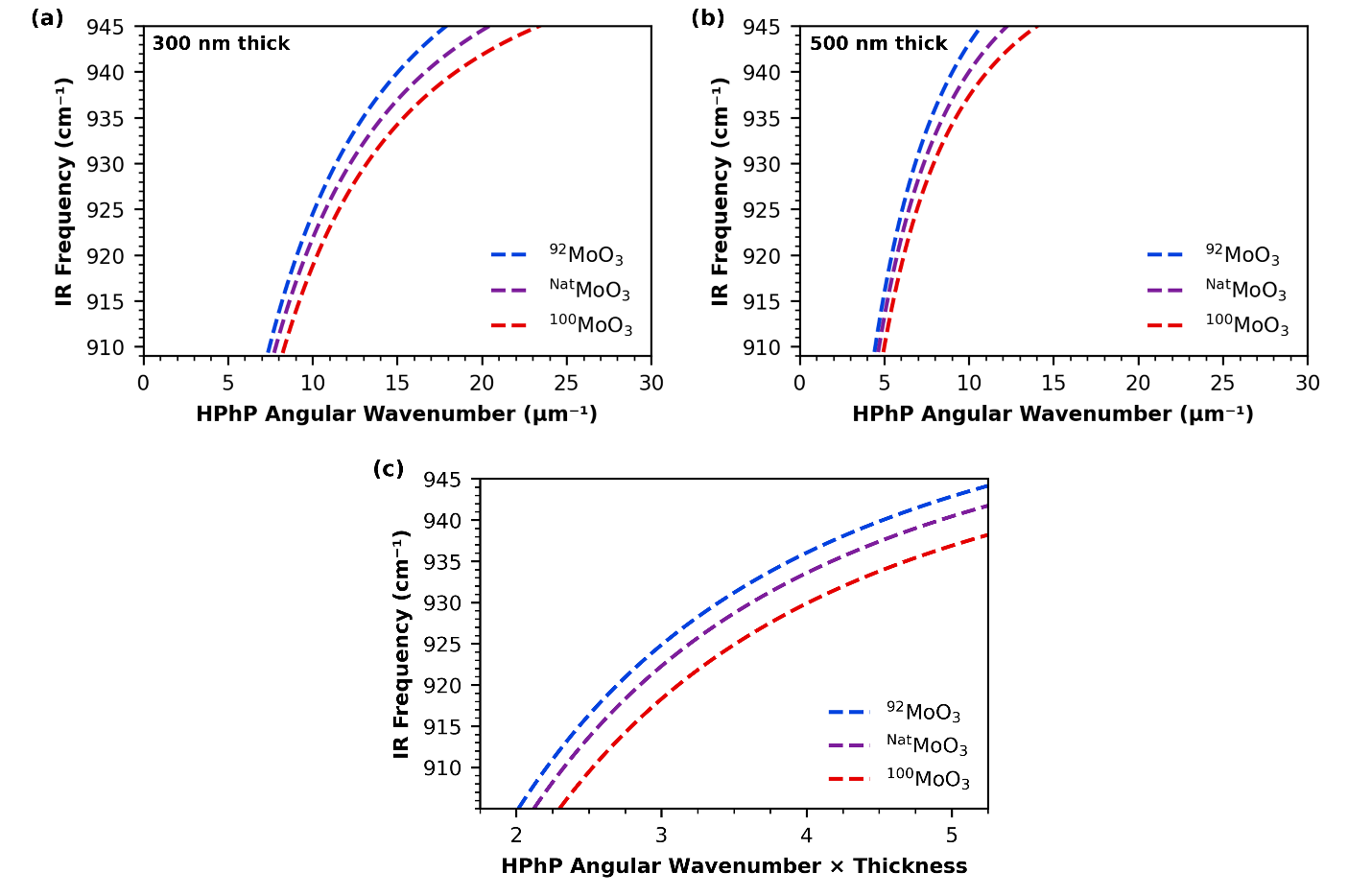
**

**Figure S6:** Effects of thickness on the theoretical hyperbolic phonon polariton (HPhP) dispersion. (a-b) The theoretical HPhP dispersion curves for natural and isotope-enriched MoO_3_ are calculated with the analytic function (**Equation 6**) and parameters derived from photothermal induced resonance maps (**Table 1**) for two thicknesses: 300 nm (a) and 500 nm (b). (c) HPhP dispersion with respect to HPhP angular wavenumber × thickness.

**Figure of Merit Comparisons**

Besides for comparing the lifetimes of HPhPs the effects of isotopic enrichment are sometimes assessed with a figure of merit (FOM) defined as [S7]:

| $\mathrm{FOM}=\frac{\mathrm{Re}(k)}{\mathrm{Im}(k)}$ | (S3) |
| --- | --- |

This can be evaluated in terms of the properties obtained by fitting the PTIR absorption profiles with **Equation S3**. Here $\lambda=\frac{2\pi}{\mathrm{Re}\left( k \right)}$ and $L_{P}=\frac{1}{2\mathrm{Im}\left( k \right)}$ [S8], where $\lambda$ is the polariton wavelength, $k$ the HPhP wavevector, and $L_{P}$ the propagation length. Taken together, and accounting for the damped harmonic oscillator model modified to be specific to tip-launched HPhP modes used here, the FOM can be expressed as:

| $\mathrm{FOM}=k\times L_{P}$ | (S4) |
| --- | --- |

where $k$ is the HPhP angular wavenumber and $L_{P}$ is the measured propagation length. The results are plotted below in **Figure S5**, where the isotopically pure samples show improvements over naturally abundant MoO_3_.


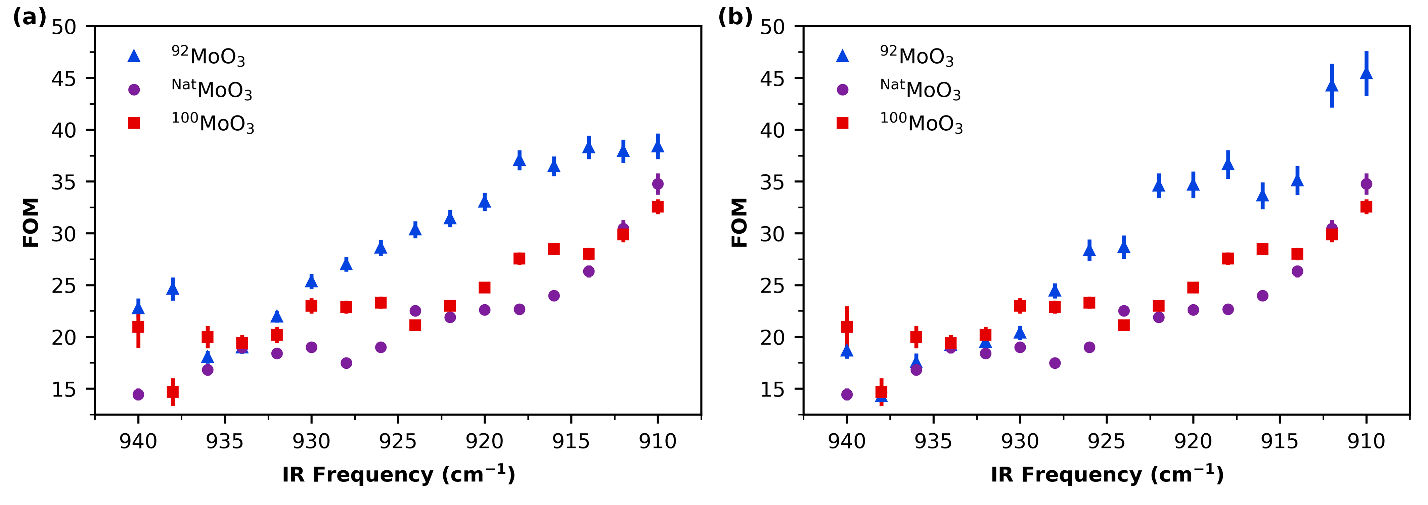


**Figure S7:** The figure of merit (FOM) of HPhPs in the different samples for ≈ 300 nm thick flakes (a) and ≈ 500 nm thick flakes (b) described in Figures 3 and S4 respectively.

**S4 Theoretical Hyperbolic Phonon Polariton Dispersion**

The dispersion of HPhPs in α-MoO_3_ crystals were predicted by and compared against two complementary theoretical analyses reported elsewhere [S9]. An analytic function **(Equation 6)** describes the complex HPhP wavenumber ($\tilde{k}$) as a function of the frequency (*ω*) of the incident IR light.

The dispersion of in-plane HPhPs that propagate along the [100] axis can also be estimated by calculating the complex Fresnel reflectance of the three-layer system, in this case air/α-MoO_3_/Au substrate [S10]. The imaginary component of the reflectance function ($r_{p}$ in **Equation (S2)**) has local maxima at wavenumbers that correspond to the HPhP modes supported by the system, which are related to energy dissipation in the hyperbolic material.

| $r_{p}= \frac{r_{1}+r_{3}e^{i2k_{2}d}}{1+ r_{1}r_{3}e^{i2k_{2}d}}$ | (S5) |
| --- | --- |

where

| $r_{1}= \frac{\varepsilon_{[100]}k_{1}-\varepsilon_{1}k_{2}}{\varepsilon_{[100]}k_{1}+\varepsilon_{1}k_{2}}$ | $r_{3}= \frac{\varepsilon_{3}k_{2}-\varepsilon_{[100]}k_{3}}{\varepsilon_{3}k_{2}+\varepsilon_{[100]}k_{3}}$ |
| --- | --- |

| $k_{j}=\sqrt{\varepsilon_{j}\left( \frac{\omega}{c} \right)^{2}-q^{2}}$ $j= 1, 3$ | $k_{2}=\sqrt{\varepsilon_{[100]}\left( \frac{\omega}{c} \right)^{2}-{\frac{\varepsilon_{[100]}}{\varepsilon_{[010]}}q}^{2}}$ |
| --- | --- |

Here, the subscripts refer to the three materials in the stack: air (1), α-MoO_3_ (2), and substrate (3). The parameter $d$ is the thickness of the flake. $r_{1}$ and $r_{3}$ correspond to the reflectivities of the air/α-MoO_3_ and α-MoO_3_/Au substrate, respectively. $\varepsilon_{j}$ $(j= 1, 3$) refers to the dielectric functions of the air and Au. $\varepsilon_{[100]}, \varepsilon_{[010]}$ refer to the dielectric functions of α-MoO_3_ along the [100] and [010] axes respectively. $k_{j}$ is the momentum component of the propagating wave along the axis.

The permittivities and phonon modes used in these calculations were first obtained from published sources (see **Table S1** and Refs. [S1, S3, S11, S12]) and then modified to reflect the observed effects of isotope enrichment. Reported values contain some degree of variability between experiment and theory, which has been attributed to potential differences in material stoichiometry and crystallinity, which can affect inter- and intra-layer interactions [S6, S13].

**
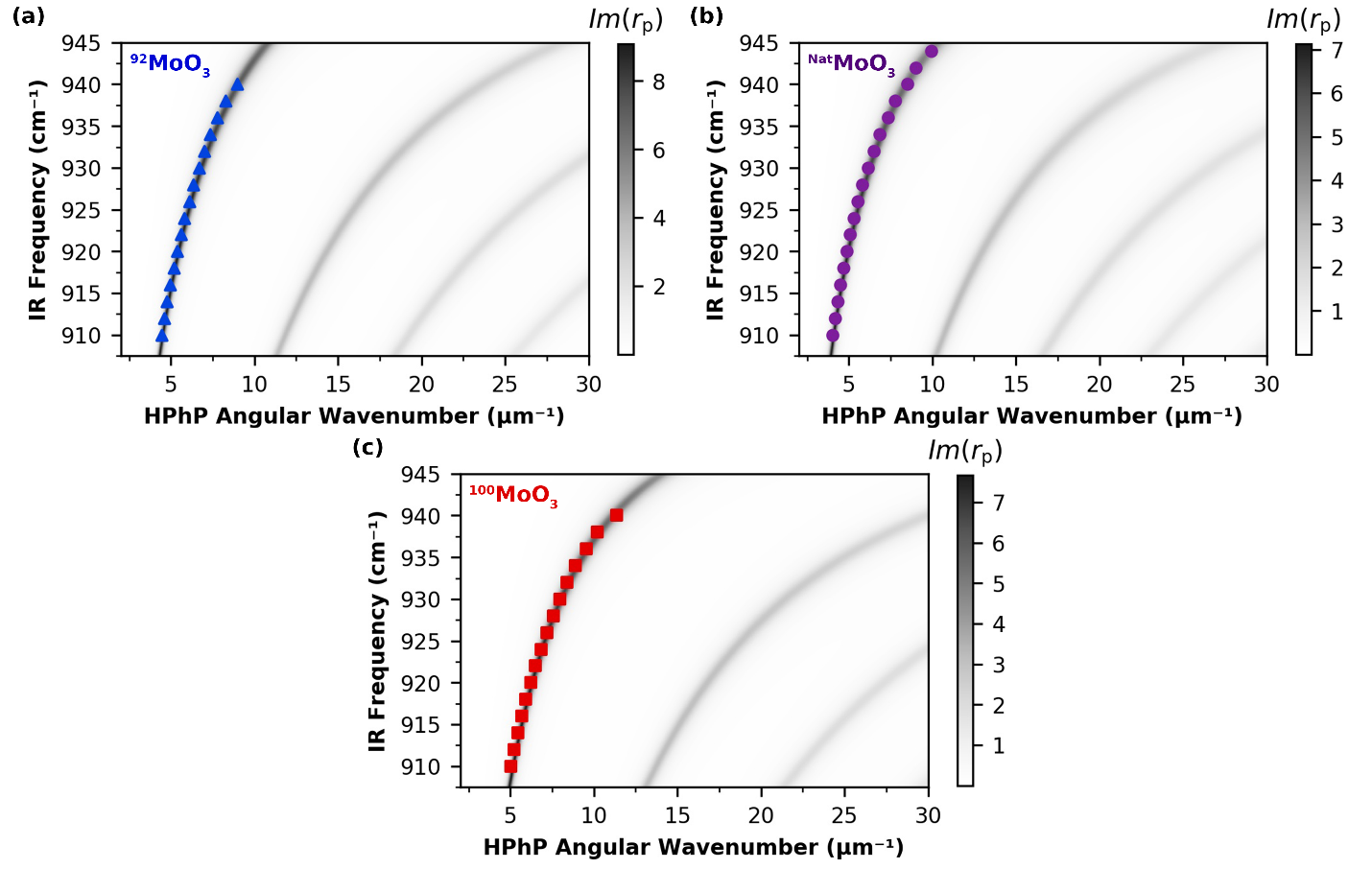
**

**Figure S8:** Comparisons of measured in-plane hyperbolic phonon polariton (HPhP) dispersions (solid symbols) to the imaginary component, $Im\left( r_{p} \right)$, of the complex Fresnel reflectance of a three-layer model system (air/MoO_3_/gold substrate). Results are presented for the ^92^MoO_3_ (a), ^Nat^MoO_3_ (b), and ^100^MoO_3_ (c) flakes characterized in **Figure S4**.

**Supplementary Material References:**

[S1] Y. Zhao, J. Chen, M. Xue*, et al.*, "Ultralow-loss phonon polaritons in the isotope-enriched α-MoO_3_," *Nano Lett.,* vol. 22, no. 24, pp. 10208-10215, 2022, <https://doi.org/10.1021/acs.nanolett.2c03742>.

[S2] Z. Zheng, N. Xu, S. L. Oscurato*, et al.*, "A mid-infrared biaxial hyperbolic van der Waals crystal," *Sci. Adv.,* vol. 5, no. 5, p. eaav8690, 2019, <https://doi.org/10.1126/sciadv.aav8690>.

[S3] G. Álvarez-Pérez, T. G. Folland, I. Errea*, et al.*, "Infrared permittivity of the biaxial van der Waals semiconductor α-MoO_3_ from near- and far-field correlative studies," *Adv. Mater.,* vol. 32, no. 29, p. 1908176, 2020, <https://doi.org/10.1002/adma.201908176>.

[S4] W. Ma, P. Alonso-González, S. Li*, et al.*, "In-plane anisotropic and ultra-low-loss polaritons in a natural van der Waals crystal," *Nature,* vol. 562, no. 7728, pp. 557-562, 2018, <https://doi.org/10.1038/s41586-018-0618-9>.

[S5] M. A. Py, P. E. Schmid, and J. T. Vallin, "Raman scattering and structural properties of MoO_3_," *Il Nuovo Cimento B (1971-1996),* vol. 38, no. 2, pp. 271-279, 1977, <https://doi.org/10.1007/BF02723496>.

[S6] M. A. Py and K. Maschke, "Intra- and interlayer contributions to the lattice vibrations in MoO_3_," *Physica B+C,* vol. 105, no. 1, pp. 370-374, 1981, <https://doi.org/10.1016/0378-4363(81)90278-3>.

[S7] A. Fali, S. T. White, T. G. Folland*, et al.*, "Refractive index-based control of hyperbolic phonon-polariton propagation," *Nano Lett.,* vol. 19, no. 11, pp. 7725-7734, 2019, <https://doi.org/10.1021/acs.nanolett.9b02651>.

[S8] S. Dai, J. Quan, G. Hu*, et al.*, "Hyperbolic phonon polaritons in suspended hexagonal boron nitride," *Nano Lett.,* vol. 19, no. 2, pp. 1009-1014, 2019, <https://doi.org/10.1021/acs.nanolett.8b04242>.

[S9] S. Dai, Z. Fei, Q. Ma*, et al.*, "Tunable phonon polaritons in atomically thin van der Waals crystals of boron nitride," *Science,* vol. 343, no. 6175, pp. 1125-1129, 2014, <https://doi.org/10.1126/science.1246833>.

[S10] Z. Zheng, J. Chen, Y. Wang*, et al.*, "Highly confined and tunable hyperbolic phonon polaritons in van der Waals semiconducting transition metal oxides," *Adv. Mater.,* vol. 30, no. 13, p. 1705318, 2018, <https://doi.org/10.1002/adma.201705318>.

[S11] J. J. Schwartz, S. T. Le, S. Krylyuk, C. A. Richter, A. V. Davydov, and A. Centrone, "Substrate-mediated hyperbolic phonon polaritons in MoO_3_," *Nanophotonics,* vol. 10, no. 5, pp. 1517-1527, 2021, <https://doi.org/10.1515/nanoph-2020-0640>.

[S12] R. L. Olmon, B. Slovick, T. W. Johnson*, et al.*, "Optical dielectric function of gold," *Phys. Rev. B,* vol. 86, no. 23, p. 235147, 2012, <https://doi.org/10.1103/PhysRevB.86.235147>.

[S13] M. Vasilopoulou, A. M. Douvas, D. G. Georgiadou*, et al.*, "The influence of hydrogenation and oxygen vacancies on molybdenum oxides work function and gap states for application in organic optoelectronics," *J. Am. Chem. Soc.,* vol. 134, no. 39, pp. 16178-16187, 2012, <https://doi.org/10.1021/ja3026906>.
